# Editorial Note: C4b Binding Protein Binds to CD154 Preventing CD40 Mediated Cholangiocyte Apoptosis: A Novel Link between Complement and Epithelial Cell Survival

**DOI:** 10.1371/journal.pone.0359124

**Published:** 2026-09-28

**Authors:** The PLOS One Editors

The *PLOS One* Editors issue this Editorial Note to inform readers that concerns have been raised about results presented in Fig 5I and Fig 5II of this article [1].
